# A Pilot Study on the Impact of Menstrual Cycle Phase on Elite Australian Football Athletes

**DOI:** 10.3390/ijerph18189591

**Published:** 2021-09-12

**Authors:** Mikaeli A. Carmichael, Rebecca L. Thomson, Lisa J. Moran, Joel R. Dunstan, Maximillian J. Nelson, Michael L. Mathai, Thomas P. Wycherley

**Affiliations:** 1 Alliance for Research in Exercise, Nutrition and Activity (ARENA), Allied Health and Human Performance, University of South Australia, Adelaide, SA 5001, Australia; r.thomson@adelaide.edu.au (R.L.T.); joel.dunstan@mymail.unisa.edu.au (J.R.D.); max.nelson@unisa.edu.au (M.J.N.); tom.wycherley@unisa.edu.au (T.P.W.); 2 Adelaide Medical School and Robinson Research Institute, Faculty of Health and Medical Sciences, University of Adelaide, Adelaide, SA 5000, Australia; 3 Monash Centre for Health Research and Implementation, School of Public Health and Preventative Medicine, Monash University, Clayton, VIC 3168, Australia; lisa.moran@monash.edu; 4 Institute for Health and Sport, College of Health and Biomedicine, Victoria University, Melbourne, VIC 8001, Australia; michael.mathai@vu.edu.au

**Keywords:** menstruation, sport, female, fatigue, wellbeing, physical performance

## Abstract

The effect of the menstrual cycle on athlete performance, wellbeing and perceived exertion and fatigue is not well understood. Furthermore, it has not been investigated specifically in Australian Football athletes. This pilot study aimed to explore how naturally menstruating Australian Football athletes may be affected by menstrual cycle phase. The data collected from the routine monitoring of five naturally menstruating athletes (average menstrual cycle length of 28 ± 3 [SD] days) in one team (athlete age range 18–35 years) competing in the Women’s Australian Football League during the 2019 season were retrospectively analysed to compare performance (countermovement jump parameters and adductor squeeze pressure), perceived exertion, perceived fatigue and wellbeing (perceived sleep quality, stress and soreness) outcomes between the follicular and luteal phases. Performance, perceived exertion, stress and soreness did not appear to be affected by menstrual cycle phase (*p* > 0.17). However, perceived fatigue appeared to be significantly greater (*p* = 0.042) and sleep quality worse (*p* = 0.005) in the luteal phase. This pilot study suggests further research focusing on the effect of menstrual cycle phase on subjective fatigue and wellbeing is warranted.

## 1. Introduction

The introduction of the Women’s Australian Football League (AFLW) is one recent contributor to the professionalisation of domestic team sports for female athletes in Australia. Since its inaugural season in 2017, there has been a surge in female participation in Australian Football (AF) across all competition levels [1] and the AFLW has expanded to include more teams competing over a longer season, with all matches in the 2021 season being ticketed for the first time [2].

AF is a demanding field-based team sport played on a large oval (approximately 165 × 135 m^2^); making it unique to other football codes such as rugby and soccer, due to the greater space afforded for athletes to move in during match-play [3]. AF is comparable to Gaelic Football, as athletes must dribble (run and bounce the ball at least once every 15 m), handball (pass the ball by punching it out of the opposite hand) or kick the ball to move it around the pitch. During the four 15 min quarters (plus stoppage time) of an AFLW match, each athlete typically spends 54 min on the field and can cover a total distance of between 5 and 7 km, of which 96 to 209 m is covered running at very high speeds (>20 kph) [4].

An emerging area of interest in AF is the effect that menstrual cycle (MC) phase may have on athletes’ performance. Many female athletes perceive a change in performance during training and competition in some phases of the MC, commonly the late luteal and early follicular phases [5,6,7]. Some studies have highlighted how menstrual-related symptoms (e.g., abdominal cramps, tiredness/fatigue) can limit athletes’ ability to train [8,9]. However, many of these studies are cross-sectional and there are very few studies that examine athletes’ perceptions of their performance, menstrual symptoms and subjective outcomes related to recovery and fatigue status in different MC phases. There is some, albeit conflicting, evidence to suggest perceived exertion and fatigue during exercise may be heightened in some MC phases [10,11,12] and delayed onset muscle soreness is increased at the start of the MC [13]. The assessment of subjective outcomes in athletes is encouraged as it identifies changes in wellbeing and athletes’ responses to training loads [14]; therefore, subjective outcomes should be examined across the MC.

Performance may fluctuate throughout the MC via hormonally mediated changes in neuroexcitation, substrate metabolism and thermoregulation, but the literature pertaining to the effects of MC phase on athletes’ performance and the mechanisms underpinning performance presents conflicting findings [15]. An extensive meta-analysis by McNulty and colleagues found the influence of MC phase on exercise performance in eumenorrheic women to be trivial, with performance being slightly reduced in the early follicular phase [16]. Studies on team sport athletes, such as soccer athletes, have demonstrated intermittent endurance performance may be reduced in the early follicular phase compared to the mid luteal phase [17], but anaerobic performance (i.e., sprints and countermovement jump) appeared to be unaffected by MC phase [17,18].

The impact of MC phase on elite AF athletes’ performance, fatigue and wellbeing has yet to be quantified, despite the continued professionalisation of the AFLW. There is currently insufficient high-quality evidence to allow practitioners to consider MC phase in the development of training and recovery programs in the AFLW and other elite team sports. Therefore, this pilot study aims to explore how MC phase may influence performance, perceived exertion, fatigue and wellbeing within AF athletes.

## 2. Materials and Methods

A retrospective, longitudinal cohort analysis was performed using routine athlete monitoring data and urine samples collected throughout the 2019 AFLW season (approximately 3 months). Ethical approval was provided by the University of South Australia’s Human Research Ethics Committee (Ethics Approval No. 200264).

### 2.1. Subjects

A convenience sample of 5 elite AF athletes from one team competing in the AFLW (age range 18–35 years) consented to their data and urine samples being analysed. The participants did not use hormonal contraception (HC) and had an MC length between 21 and 35 days, as determined by the MC diaries. One participant commenced using HC during the data collection period. They were included in the study, but their data were truncated to exclude values from the day they reported commencing HC use.

### 2.2. Menstrual Cycle Phase Identification

The follicular and luteal MC phases were identified using MC diaries and verified, where possible, using urine sample analysis. The MC diary was completed daily using a symbol entry to capture when spotting, light, moderate or heavy bleeding occurred. The MC diaries determined the onset of the follicular phase (i.e., first day of menstrual bleeding) and conclusion of the luteal phase (i.e., day prior to menstrual bleeding beginning). The luteal phase was estimated to have started 14 days prior to the onset of menstrual bleeding, as the luteal phase is typically more consistent in duration than the follicular phase [19]. Where a participant reported spotting in the days leading up to menstrual bleeding, the onset of the follicular phase was considered to be when bleeding, not spotting, first occurred [20].

Participants were instructed to collect 5 to 10 millilitre spot urine samples every second day from the mid-stream of the first morning micturition as a method of verifying MC phase. The estimated onset of the luteal phase was adjusted, when sufficient urine sample data were available (42% of points of ovulation were changed), if urinary pregnanediol glucuronide (PdG) levels indicated there was a sustained post-ovulatory progesterone surge that was inconsistent with the 14-day prediction. Participant compliance with urine sample collections was moderate, as only 66% of scheduled urine samples were collected (and only 3 participants collected at least 75% of schedule samples). PdG was determined using a competitive enzyme-ligand conjugate immunoassay [21]. PdG concentration in the urine samples was measured and converted to a secretion rate using specific gravity as an estimate of urinary excretion rate [22].

### 2.3. Outcome Measures

The following outcome measures were analysed: countermovement jump and adductor squeeze tests, a wellness questionnaire and rating of perceived exertion. These measures were undertaken during the routine athlete monitoring program at the football club and have been previously used or validated in research studies as markers of physical performance, perceived exertion, fatigue and wellbeing.

#### 2.3.1. Countermovement Jump

A single countermovement jump (CMJ) was performed on a Kistler Force Plate (Kistler Instruments Australia Pty Ltd., Wheelers Hill, Australia) prior to the first training session each week. Participants were encouraged to jump as high as possible whilst holding a dowel across their shoulders to negate the influence of arm swing and self-select the countermovement squat depth. The performance outcomes recorded were peak power (W), peak relative power (W/kg) and flight to contraction time ratio. 

#### 2.3.2. Adductor Squeeze

The adductor squeeze test was undertaken following the CMJ, with an inflated (10 mmHg) sphygmomanometer cuff placed between the knees, proximal to the medial femoral epicondyles. Participants performed a maximal effort squeeze with legs straight (0° knee flexion) and bent (60° knee flexion) whilst in a supine position. Performance was recorded as the peak pressure (mmHg) exerted in each position. 

#### 2.3.3. Wellness Questionnaire

**Figure 1:**
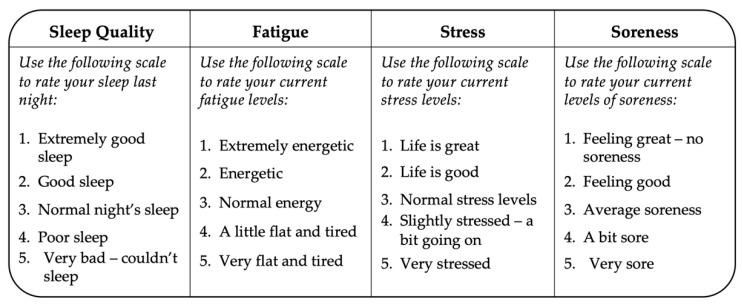
Wellness questionnaire, adapted from McLean and colleagues [23].

Wellbeing and fatigue were measured using a wellness questionnaire, adapted from McLean and colleagues [23]. The questionnaire (Figure 1) was completed prior to every training session, approximately twice per week. It comprised of four questions related to perceived sleep quality, stress, soreness and fatigue.

#### 2.3.4. Perceived Exertion

A rating of perceived exertion (RPE) value was reported immediately following each training session, using the modified CR-10 Scale [24]. Training session duration was not recorded, and training load could therefore not be determined; hence, the RPE value provides a subjective marker of intensity during a training session, where notable differences may indirectly reflect an athlete’s level of fatigue.

### 2.4. Statistical Analysis

The data were analysed using statistical software package SPSS Version 25 (IBM Corp, Armonk, NY, USA). Descriptive statistics for each outcome in both MC phases were calculated and reported using means and standard deviations (SD). Normality of the data was checked prior to analysis. Linear mixed models were used to investigate the within participant differences between the performance, perceived exertion, fatigue and wellbeing outcomes by MC phase. The significance threshold was set to *p* < 0.05. Due to the small sample size and subsequent preliminary nature of the analysis, correction for multiple analyses was not performed for this study. The raw data figures (non-mixed model figures) are reported for the absolute and change values and the *p*-values are reported from the linear mixed model. A supplementary analysis comparing performance, fatigue and wellbeing outcomes during menstruation vs. the rest of the cycle is included in Table A1, in Appendix A.

## 3. Results

The average (±SD) MC length of the participants was 28 ± 3 days. The results comparing performance, perceived exertion, fatigue and wellbeing between the two MC phases are presented in Table 1.

The objective performance (CMJ and adductor squeeze) outcomes did not differ between the MC phases (*p* > 0.17). Perceived exertion, stress and soreness were also unaffected by MC phase (*p* > 0.22). Sleep quality (*p* = 0.005) and fatigue (*p* = 0.042) were worse during the luteal phase. 

## 4. Discussion

This pilot study was the first to explore how elite AF athletes’ performance, perceived exertion, fatigue and wellbeing may be affected by MC phase. The main finding of this pilot study was that AF athletes might experience a decline in subjective sleep quality and heightened subjective fatigue in the luteal phase of the MC. Both sleep quality and fatigue influence athletes’ health and readiness to train and compete [25]. Whilst there is high interest in sleep quality in sport science, due to its established link to recovery and performance [26], there is a paucity of research on MC-based changes in sleep within athletes. During menstrual bleeding and the follicular phase, female athletes spend more time in bed and more time in slow wave (deep) sleep compared to the luteal phase [27], which is in agreement with the preliminary findings in AFLW athletes in the current study. Home electroencephalography has demonstrated that female collegiate athletes had a significantly lower total sleep time, sleep efficiency and prolonged sleep latency during the first early follicular phase compared to the mid follicular phase. Whilst this study did not consider sleep during the luteal phase, it does highlight a difference in sleep quality in different phases of the MC and suggests that decreases in sleep quality were exacerbated by the occurrence of menstrual symptoms [28]. In non-athletic populations, sleep quality, indicated by self-reported and objective measures, has also been found to be poorer in the late [29] and mid luteal phases [30], respectively. The diminished sleep quality in the luteal phase may be a consequence of the increase in core body temperature when progesterone is elevated [29,30].

There was no observed effect of MC phase on performance (adductor squeeze and countermovement jump outcomes) in this study. This finding and the results from the recent meta-analysis by McNulty and colleagues [16] suggests MC phase is unlikely to be a substantial factor underpinning the strength- and power-based physical performance capacity of AF athletes. This is despite it being mechanistically feasible for fluctuations in strength and power across the MC to occur from progesterone inhibiting cortical excitability and force production [31] and the neuroexcitatory effect of oestrogen enhancing strength [31,32,33,34]. Theoretically, strength and power would decrease in the luteal phase, as progesterone is elevated, but improve in the late follicular phase, as oestrogen peaks. However, in studies citing an effect of the MC on jump performance, the phase that demonstrated peak performance has been inconsistent [35,36,37]. It is possible that the null-findings for the performance outcomes observed in the current study may be a result of the small sample size and/or the bi-phasic MC model utilised, which did not allow an analysis of specific MC sub-phases (i.e., early and late follicular phases) to be conducted. Additionally, this study on AFLW athletes did not measure endurance performance, which, in soccer athletes, has been influenced by MC phase [17]; this should be considered in future research.

The trend for perceived fatigue to be greater in the luteal phase could be explained by the lower sleep quality also reported in the luteal phase. Fatigue is also a common menstrual-related symptom that can be experienced during the late luteal phase [38]. Li, Lloyd and Graham [39] found that mental fatigue in eumenorrheic individuals was also increased during the mid-luteal phase, when the progesterone concentration typically peaks, compared to the early follicular phase, but physical fatigue remained unchanged. In a cross-sectional survey of recreational to elite Australian athletes, approximately half of respondents believed their ability to train and compete was negatively impacted by their MC and the most common reasons cited were feeling that they “fatigue more easily” or their “energy levels are different” [5]. It was also reported that athletes self-reporting heavy menstrual bleeding are more likely to report having menstrual symptoms related to fatigue [5]; it is possible it may have a role in some athletes’ perceptions of fatigue as a significant link between the experience of heavy menstrual bleeding, perceptions of fatigue, and haemoglobin and ferritin levels has already been established in general population reproductive aged females [40].

In this pilot study, MC phase appeared not to impact on perceived stress in AF athletes. Perceived stress in athletes throughout the MC has not been well-studied, but in non-athletes, it is generally reported to be greater in the late luteal phase [41]. The increased perception of stress may be caused by oestrogen’s role in the metabolism of serotonin, where the reduced oestrogen concentration allows for increased serotonin metabolism, and lower levels of serotonin consequently have a negative affect [41]. This mechanism could feasibly contribute to heightened stress or a depressed mood in athletes during the late luteal and early follicular phases when oestrogen remains low. The bi-phasic MC model used in this study on AFLW athletes did not enable the identification of fluctuations that may have occurred within sub-phases of the follicular and luteal phases; this could be considered in future research. 

Perceived soreness and exertion appeared to be unaffected by MC phase. This is in contrast with the findings of previous studies that have found, under standardised conditions, muscle soreness before [42] and immediately post exercise [43] is greatest in the early follicular phase. However, delayed onset muscle soreness (24 and 48 h post-exercise) was not significantly affected by MC phase [43]. Perceived exertion has also been found to be either unaffected by MC phase [11] or greater in the follicular phase, compared to the luteal phase, in active [44] and well-trained [45] populations. Nevertheless, the lack of an MC phase effect on these AF athletes could be present, as factors known to influence muscle soreness, including the intensity or type of exercise (eccentric or concentric), recovery time and interventions to reduce symptoms of muscle damage, were not considered. Other confounders that could affect the rating of perceived exertion, including prescribed training loads and environmental heat stress, were unable to be assessed in this study on AF athletes.

There are several limitations in this study. The major limitation was the small sample size that resulted from using retrospective data from athletes in just one team, across one AFLW season. The bi-phasic MC model was also a limitation. Ideally, performance, fatigue and wellbeing would be investigated in specific subphases of the MC, such as the early follicular, ovulatory or mid luteal phases, that possess unique female sex hormone profiles [46]. The follicular and luteal phases were examined due to the observational and pragmatic nature of this pilot study; consistent data were unavailable to enable an investigation of additional MC subphases.

Another limitation of this study is the lack of consideration for menstrual symptoms. Many athletes and active females experience a variety or menstrual symptoms at varying degrees of severity and these symptoms can impact an individual’s ability to train, work or go to school [8,9]. Menstrual symptoms may also impact the extent to which an individual’s performance, fatigue or wellbeing is affected by their MC, as early follicular declines in sleep quality are exacerbated in athletes experiencing more severe menstrual symptoms [28]. Future research should include outcome measures related to the type, severity and timing of menstrual symptoms.

The method of MC phase identification used also limited the consistent and accurate identification of the onset of the luteal phase. In this study, there was only moderate compliance with urine sample collection (66% of scheduled urine samples were collected); therefore, the counting method that was primarily used to determine MC phase in this study was not always able to be conclusively validated with urine PdG levels. 

Nevertheless, this study was highly novel, being the first to specifically examine AF athletes, a unique cohort participating in a rapidly growing sport in Australia, that has a relatively small evidence base relative to male AF athletes. It fulfilled its aim to explore the present trends to inform the directions of future research. In terms of hypothesis generation, these results suggest naturally menstruating AF athletes may be susceptible to cyclic variations in subjective sleep quality and fatigue.

The reduced sleep quality in the luteal phase and the recently reported finding that elite female AF athletes experience significantly lower quality sleep than their male counterparts [47] highlights that further research on sleep quality in female athletes is warranted. Laboratory- and non-laboratory-based sleep assessment tools should be used to provide objective measures of sleep quality throughout the MC. If these findings are substantiated, the effectiveness of interventions to improve sleep quality in the luteal phase should also be investigated.

It is important the impact of MC phase on fatigue is further researched, given many AFLW athletes still continue to work full or part-time during the AFLW season [48]. Subjective and objective markers of fatigue, such as self-reports and cardiovascular or biochemical responses to exercise, should be investigated in various MC phases to confirm whether athletes are at risk of heightened fatigue during the luteal phase. 

## 5. Conclusions

High quality research that utilises large sample sizes, considers the MC sub-phases and adheres to the most recent guidelines for research on female athletes [46] is necessary to further examine performance, fatigue and wellbeing, and validate the preliminary findings of the current study. 

In the absence of conclusive results, sport science practitioners in the AFLW and other team sports should follow the most recent guidelines outlined in an Expert Statement from the British Association of Sport and Exercise Sciences to implement MC monitoring for athletes that believe they are affected by their MC, before modifying training, recovery or nutrition programs on an individual basis after identifying any trends that may be addressed [49]. 

The results from this study, although preliminary, suggest sleep quality and fatigue, factors that are significant to health and athlete readiness [25], might be impacted by MC phase in elite AF athletes.

## Figures and Tables

**Table 1:** Performance, fatigue and wellbeing outcomes in the follicular and luteal menstrual cycle phases.

	Follicular		Luteal		
Outcome	*n*	Mean ± SD	*n*	Mean ± SD	P
AS 0° (mmHg)	15	196 ± 48.1	21	201.4 ± 48	0.362
AS 60° (mmHg)	15	228 ± 58.8	21	230 ± 47.9	0.816
CMJ FT: CT	4	0.53 ± 0.09	7	0.48 ± 0.04	0.173
CMJ PP (W)	4	2850.7 ± 280.2	7	2812 ± 135.2	0.510
CMJ RPP (W/kg)	4	43.2 ± 8.3	7	42 ± 4.8	0.264
Fatigue *	17	3.24 ± 0.56	28	3.68 ± 0.82	0.042
Sleep *	17	2.65 ± 0.86	28	3.54 ± 1.11	0.005
Soreness	17	2.65 ± 0.79	28	2.82 ± 0.82	0.262
Stress	17	3.12 ± 0.33	28	3.32 ± 0.48	0.224
RPE	25	3.36 ± 1.29	30	3.37 ± 1.52	0.670

AS 0° (mmHg) = Adductor squeeze peak pressure (mmHg) at 0° of knee flexion; AS 60° (mmHg) = Adductor squeeze peak pressure (mmHg) at 60° of knee flexion; CMJ FT: CT = Countermovement jump flight to contraction time ratio; CMJ PP (W) = Countermovement jump absolute peak power (W); CMJ RPP (W/kg) = Countermovement jump relative peak power (W/kg); *n* = Number of times the outcome was measured; P = *p*-value for between group difference; RPE = Rating of perceived exertion; SD = Standard deviation. * Significant difference between menstrual cycle phases.

## Data Availability

The raw data supporting the conclusions of this article will be made available by the authors upon reasonable request.

## Appendix A

**Table A1:** Performance, fatigue and wellbeing outcomes during menstruation and non-menstruation cycle.

	Menstruation		Non-Menstruation		
Outcome	*n*	Mean ± SD	n	Mean ± SD	P
AS 0° (mmHg)	3	160 ± 20	33	202.73 ± 47.72	0.99
AS 60° (mmHg)	3	193.33 ± 80.83	33	232.42 ± 49.06	0.62
Fatigue	7	3.43 ± 0.79	38	3.53 ± 76	0.64
Sleep	7	2.57 ± 1.13	38	3.32 ± 1.07	0.07
Soreness	7	2.29 ± 0.49	38	2.84 ± 0.82	0.08
Stress	7	3.29 ± 0.49	38	3.24 ± 0.43	0.71
RPE	9	3.44 ± 1.33	46	3.35 ± 1.43	0.81

AS 0° (mmHg) = Adductor squeeze peak pressure (mmHg) at 0° of knee flexion; AS 60° (mmHg) = Adductor squeeze peak pressure (mmHg) at 60° of knee flexion; CMJ FT: CT = Countermovement jump flight to contraction time ratio; CMJ PP (W) = Countermovement jump absolute peak power (W); CMJ RPP (W/kg) = Countermovement jump relative peak power (W/kg); *n* = Number of times the outcome was measured; P = *p*-value for between group difference; RPE = Rating of perceived exertion; SD = Standard deviation.
